# Randomized Controlled Trial of Adding Telephone Follow-Up to an Occupational Rehabilitation Program to Increase Work Participation

**DOI:** 10.1007/s10926-017-9711-4

**Published:** 2017-06-09

**Authors:** Karen Walseth Hara, Johan Håkon Bjørngaard, Søren Brage, Petter Christian Borchgrevink, Vidar Halsteinli, Tore Charles Stiles, Roar Johnsen, Astrid Woodhouse

**Affiliations:** 1 Department of Public Health and Nursing, Faculty of Medicine and Health Sciences, Norwegian University of Science and Technology (NTNU), Postbox 8905, 7491 Trondheim, Norway; 2 Norwegian Advisory Unit on Complex Symptom Disorders, St. Olavs Hospital, Trondheim University Hospital, Trondheim, Norway; 3 Department of Circulation and Medical Imaging, Faculty of Medicine and Health Sciences, Norwegian University of Science and Technology (NTNU), Trondheim, Norway; 4 The Norwegian Labour and Welfare Service of Sør-Trøndelag, Trondheim, Norway; 5 Forensic Department and Research Centre Brøset, St. Olavs Hospital, Trondheim University Hospital, Trondheim, Norway; 6 The Norwegian Directorate for Labour and Welfare, Oslo, Norway; 7 Hysnes Rehabilitation Center, St. Olavs Hospital, Trondheim University Hospital, Trondheim, Norway; 8 Centre for Health Care Improvement, St. Olavs Hospital, Trondheim University Hospital, Trondheim, Norway; 9 Department of Psychology, Faculty of Social and Educational Sciences, Norwegian University of Science and Technology (NTNU), Trondheim, Norway

**Keywords:** Acceptance and commitment therapy, Mental disorders, Musculoskeletal pain, Vocational rehabilitation, Telerehabilitation

## Abstract

*Purpose* Transfer from on-site rehabilitation to the participant’s daily environment is considered a weak link in the rehabilitation chain. The main objective of this study is to see if adding boosted telephone follow-up directly after completing an occupational rehabilitation program effects work participation. *Methods* A randomized controlled study included participants with chronic pain, chronic fatigue or common mental disorders on long-term sick leave. After completing 3½ weeks of acceptance and commitment therapy based occupational rehabilitation, participants were randomized to boosted follow-up or a control group before returning to their daily environment. The intervention was delivered over 6 months by on-site RTW coordinators mainly via telephone. Primary outcome was RTW categorized as participation in competitive work ≥1 day per week on average over 8 weeks. *Results* There were 213 participants of mean age 42 years old. Main diagnoses of sick leave certification were mental disorders (38%) and musculoskeletal disorders (30%). One year after discharge the intervention group had 87% increased odds (OR 1.87, 95% confidence interval 1.06–3.31, p = 0.031), of (re)entry to competitive work ≥1 day per week compared with the controls, with similar positive results for sensitivity analysis of participation half time (≥2.5 days per week). The cost of boosted follow-up was 390.5 EUR per participant. *Conclusion* Participants receiving boosted RTW follow-up had higher (re)entry to competitive work ≥1 day per week at 1 year when compared to the control group. Adding low-cost boosted follow-up by telephone after completing an occupational rehabilitation program augmented the effect on return-to-work.

**Electronic supplementary material:**

The online version of this article (doi:10.1007/s10926-017-9711-4) contains supplementary material, which is available to authorized users.

## Background

Modern welfare states are alarmed by high expenditure for sickness benefits and marginalization from the work force. Extensive research has been directed at designing interdisciplinary occupational rehabilitation programs. Studies are recommended to build on current evidence, to refine and increase generalizability of existing programs [1] while bearing in mind rehabilitation costs.

Obstacles to implementing a continuous chain of care between medical rehabilitation and the phase of (re)entry to competitive work have been studied [2, 3]. Transition from specialized on-site occupational rehabilitation programs to off-site, community-based return-to-work (RTW) efforts are targeted as a weak link in the rehabilitation chain [2, 3]. Strengthening individual follow-up during the phase of (re)entry to ordinary employment has been shown to increase work participation [4–7]. Integrating the efforts of health and work services is considered a critical factor for the effectiveness of RTW programs targeting both mental disorders [7] and musculoskeletal and other pain-related conditions [8]. Active involvement of a RTW coordinator is identified as a key element in successful RTW programs [8, 9]. Within the field of telerehabilitation post-discharge telephone support has been shown to be efficient in cardiac and other types of rehabilitation [10, 11], but evidence is lacking for occupational rehabilitation and the outcome of return to work.

The appropriate therapeutic approach in individual follow-up has not been strictly defined [4, 12]. Cognitive behavioral therapy (CBT) is a core component in many occupational rehabilitation programs [13, 14] and has been shown to increase RTW when integrated with individual job support [15]. Booster sessions have been shown to augment the effect of CBT. Acceptance and commitment therapy (ACT), third generation cognitive therapy, is a treatment across diagnoses [16–18] recommended for booster sessions [19, 20]. Research on booster sessions to extend occupational rehabilitation programs is scarce and has to our knowledge not been studied for programs based on ACT.

Various follow-up regimes have been implemented after multidisciplinary rehabilitation but consensus is lacking on recommended content, duration and intensity [21]. Discrepancy in content and application of components of rehabilitation programs is a well-recognized problem [1]. This pragmatic randomized controlled trial singles out post-discharge follow-up by telecommunication to investigate its particular effect as an intrinsic part of a novel occupational rehabilitation program initiated by the Norwegian government in 2012. The core component of the follow-up regime is booster sessions of ACT delivered by telephone on a monthly base.

The primary aim of this study is to explore if adding boosted RTW follow-up by telephone after an occupational rehabilitation program has an effect on work participation over the first year for individuals on long-term sick leave. The secondary aim is to calculate the added cost of boosted RTW follow-up from the perspective of the occupational rehabilitation institution.

## Methods

### Trial Design

The effectiveness of boosted follow-up versus standard RTW follow-up after on-site occupational rehabilitation is investigated in a pragmatic randomized controlled study designed to maximize applicability and generalizability of the results to usual care settings. Reporting is according to the CONSORT guidelines of 2010 [22]. The study is registered in ClinicalTrials.gov (No.: NCT01568970).

### Participants

Participants completing a 3½ week occupational rehabilitation program at Hysnes Occupational Rehabilitation Center, a rural division of Trondheim University Hospital, were consecutively invited to participate in the study from January 2012 to June 2013. Participants were referred by general practitioners (GPs) or other medical specialists and were prior to admission assessed by an interdisciplinary team at the associated outpatient department.

### Eligibility and Exclusion Criteria

Eligible participants were of age 18–59 years, referred for musculoskeletal or other chronic pain disorders, chronic fatigue or a common mental disorder and currently receiving temporary medical benefits due to work incapacity (duration over 8 weeks, partial or full-time). In Norway this involves being on one of two benefits that both require sickness certification; either sickness benefit (compensates for loss of income for employees or others with equivalent rights earned through previous participation in paid work) or work assessment allowance (for those who have either already received sickness benefits for the maximum period of 52 weeks, or have not earned the right to sickness benefits through previous employment). Participants should have a self-defined goal of increasing participation in competitive work, be adequately treated for health problems demanding acute care, be able to communicate in Norwegian and to maintain basic daily care for themselves during a stay at the rehabilitation center. Exclusion criteria were severe mental illness (ongoing mania, psychosis or suicidal ideation), active substance abuse and addiction, pregnancy, planning to enter/return to studies rather than competitive work, incomplete study registration procedure, not registered as receiving temporary medical benefits, or not completing the rehabilitation program due to acute injury/disease or personal/family reasons.

### Study Setting

The follow-up intervention took place in the participant’s daily environment directly after completing a 3½ week on-site occupational rehabilitation program. The on-site program included individual and group sessions of ACT integrated with mental and physical training and work-related problem solving. Pairs of RTW coordinators were in charge of coordinating and executing the on-site program for groups of maximum 8 participants. Activities were organized around 6–7 h “workdays” with weekends free. Core concepts of ACT were incorporated into the different components of the program; group discussions, individual meetings, physical activities, psychoeducational, and mindfulness sessions. Collaboration with community stakeholders (GPs, work place and the social security office) was initiated on-site and participants had prepared their own action plan for RTW with guidance from on-site RTW coordinators and community stakeholders. The on-site program is further described elsewhere [23].

### Intervention

Two different regimes of post-discharge RTW follow-up were compared: boosted RTW follow-up added to standard RTW follow-up (intervention group) versus standard RTW follow-up only (control group).

#### Standard RTW Follow-Up

All participants received standard community-based RTW follow-up for persons on temporary medical benefits according to national guidelines. Standard follow-up consisted of individualized follow-up delivered by different community stakeholders with predefined roles and obligations according to Norwegian legislation [24]. The social security officer acts as administrative coordinator and the GP, employer and others attend on request. This study did not register details on how standard RTW follow-up was delivered.

#### Boosted RTW Follow-Up

In addition to standard follow-up, participants randomized to the intervention group received boosted RTW follow-up. This was delivered over 6 months by the on-site RTW coordinator who after close interaction with the participant through 3½ weeks was well informed about the participant’s personal action plan for RTW, self-perceived barriers for RTW as well as any progress or changes that had occurred during on-site rehabilitation. The content of boosted RTW follow-up was in accordance with the same ACT principles that had guided on-site rehabilitation, thus providing a prolongation of therapy into the phase of reentering work. Furthermore, emphasis was on supporting graded progression towards stable return-to-work. ACT encourages value-based behavioral choices and committed actions [25] and involved strategies that participants could use in all phases of return to work, as well as other dimensions of daily life. Monthly contact was advised, but could be more frequent if the RTW coordinator and the participant found this necessary. In addition, it was recommended that RTW coordinators collaborate directly with local stakeholders. Contact was primarily by telephone, but videoconference or face-to-face meetings were other options. Booster sessions were to be continued according to the same basic principles and standard protocol for the full 6 months regardless of whether or not the participant had returned to work. The intervention could be stopped prior to 6 months if requested by the participant but such a discontinued intervention was not equivalent with withdrawal from the study. The follow-up period could be extended by 4 weeks if the RTW coordinator was prevented from delivering a continuous 6-month intervention. If the RTW coordinator was permanently prevented from delivering booster sessions the follow-up responsibility was transferred to another coordinator.

For the control group no further contact with the on-site RTW coordinator was planned. If participants in the control group or their stakeholders specifically requested contact, medico-ethical considerations had precedence, but contact was to be restricted to a minimum of clarifying conversations.

#### Adherence

The study protocol for boosted RTW follow-up was described in an intervention manual and introduced to all RTW coordinators. Adherence to study guidelines was monitored through regular interviews conducted by a researcher. RTW coordinators had various professional backgrounds (health and non-health related) and clinical adherence was ensured by an experienced clinical psychologist providing introductory training in ACT therapy and biweekly group-counseling for RTW coordinators delivering ACT therapy in the occupational rehabilitation program.

### Data Collection

Participants filled in internet-based self-report questionnaires prior to entering the rehabilitation program. On-site RTW coordinators reported from interventional contacts with participants and stakeholders. Information on participant benefits, employment state and working hours were retrieved from the database of the Norwegian Labour and Welfare Service. This included both medical (sick leave benefits, work assessment allowance, permanent disability benefits) and non-medical benefits (unemployment benefits, parenting benefits and social benefits). National modes for tracking working hours depended on the type of benefit received. The number of hours of paid work per week was collected from 6 weeks prior to entering and up to 56 weeks after completing the rehabilitation program.

### Primary Outcome

The primary outcome was (re)entry to the ordinary work force analyzed from time of discharge (randomization) through 1 year (56 weeks). The primary outcome variable was dichotomous and defined as participation in competitive work ≥1 day (7.5 h) per week on average over 8 weeks. A consensus team consisting of experts from The Norwegian Labour and Welfare Service concluded that a transition from under 7.5 h weekly work up to one full working day per week on average over 8 weeks was substantial enough to be considered a meaningful “first step” towards entering the ordinary work force. This was considered a relevant cut off for individuals with an expected poor prognosis for work. For sensitivity analysis, and to investigate increase in work participation among participants with a stronger connection to the work force, the outcome measures “minimum half-time work”, defined as ≥2.5 days (18.75 h) per week on average over 8 weeks, and “full-time work”, defined as ≥4 days (30 h) per week on average over 8 weeks, were used as more robust measures of (re)entry to work.

### Secondary Outcomes

#### Days of Paid Work

The total number of days worked (continuous measure) over the first year was used as a cumulative measure of work productivity.

#### Medical Benefits

Recipient status for temporary wage compensation benefits (medical and non medical, partial and full) was compared at 1 year after randomization.

#### Cost of Boosted RTW Follow-Up per Participant

Cost per participant was calculated by aggregating contact costs. For each contact type the number of contacts was multiplied by the accompanying unit price. Data on number of individual and collaborative contacts, and type of contact (telephone call, videoconference, face-to-face meeting) were registered for each participant. For each type of contact a fixed duration in hours was set based on recall and agreement between RTW coordinators. The unit prices or cost per contact type were calculated by multiplying the fixed number of hours by wage cost per hour. The latter was calculated from average wage for the actual RTW coordinators and included payroll tax and overhead (20%). In addition wage cost per hour were adjusted for administrative tasks assumed to account for 20% of total working hours for RTW coordinators. Costs were reported in Euros (year = 2014) and an exchange rate of 8.4 NOK per EUR^1^ was applied. Costs for the standard RTW follow-up program were not included because such contacts were assumed equally distributed across the intervention and control group.

### Descriptive Variable(s)

Baseline self-reported data and clinical data were collected prior to entering the program and used to describe the population demographically and clinically. Baseline differences between the intervention groups are reported and considered with regard to prognostic importance of the variable and size of chance imbalances.

#### Chronic Pain

Chronic pain was measured using a single question from the Short Form 8*: “How much bodily pain have you had the last week?” The response alternatives are: none, very mild, mild, moderate, severe, and very severe [26]. This scale has been validated and used as a proxy measure of chronic pain in Norwegian population studies, using a cut-off at ≥moderate pain [27]. Chronic pain was defined as duration of 6 months or more [27] and checked through assessment of clinical reports.

#### Chronic Fatigue

Chronic fatigue was measured using the 13-item Chalder Fatigue Scale [28]. Each item has four response categories, which are bimodaly scored as 0-0-1-1 (better than usual = 0, no more than usual = 0, worse than usual = 1, much worse than usual = 1). The first 11 items reflect symptom intensity. The cut-off was set at a score of ≥4 combined with symptom duration of 6 months or more. This is the recommended cut-off when using the 13-item Chalder Fatigue Scale that has been validated for a Norwegian population [29]. Likert scores were also calculated.

#### Mental Distress

Mental distress was measured using the 14-item Hospital Anxiety and Depression Scale (HADS) [30]. The HADS is made for evaluating mental distress in populations with physical symptoms. It consists of both an anxiety and a depression scale, each having seven items. Every item has four response categories from 0 to 3, giving a maximum score on each scale of 21. Mental distress is defined as present if a score ≥8 on either the anxiety and/or the depressive scales [31]. The cut-off is validated for a Norwegian population [32].

#### Sleep Disturbance

Sleep disturbance was measured using the 7-item Insomnia Severity Index (ISI). The ISI measures the nature, severity and impact of insomnia symptoms the past 2 weeks. Each item has 5 response categories from 0 to 4, giving a maximum score of 28. A cut-off of ISI ≥ 11 [33] is recommended to identify patients with clinically significant insomnia. The ISI has been shown to have very good reliability and validity [34, 35] and is recommended as an outcome measure for insomnia in clinical trials [36].

#### Self-Reported Disability

Self-reported disability was assessed using the 39-item Norwegian Function Assessment Scale (NFAS). The NFAS measures (dis)ability in seven functional domains; walking/standing; holding/picking things up; lifting/carrying, sitting, managing, cooperation/communication and vision/hearing. Each item has five response categories (no difficulty, little difficulty, moderate difficulty, much difficulty, could not do it). The NFAS was derived from the activities/participation dimension of the International Classification of Functioning, Disability and Health (ICF). It is used to assess the need for rehabilitation, adjustment of work demands among sick-listed persons and rights to social security benefits. The NFS has been validated for use in a Norwegian population [37, 38], and is shown to discriminate between individuals who are expected to report different levels of disability. A cut-off of at 3 and higher (“moderate disability” and more) is used in this study. NFAS starts with the question “Have you had difficulty with doing the following activities during the last week?” [39].

#### Main Diagnose of Sickness Certification

Data from the Norwegian Labour and Welfare Service was used to categorize the main health related cause of sick leave as reported by the treating doctor, usually the GP. The international classification of primary care (ICPC-II) was used [40].

### Sample Size

Sample size was calculated on the assumption that 50% of the control group and 70% of the intervention group would return to work within 12 months after completing the rehabilitation program. With a significance level of 5%, 186 participants would be needed to have a power of 80%. Allowing for <10% drop out, sample size was set at 200 participants. Since comparative studies on boosted RTW follow-up after occupational rehabilitation were lacking best judgment was used to make assumptions on levels of RTW.

### Randomization

An external research service performed computer-generated block randomization (block size four) with stratification according to care-provider (RTW coordinator). Randomization occurred the day before departure from the rehabilitation center to ensure that on-site rehabilitation was common for both groups. Blinding of participants and RTW coordinator to the allocation result was not feasible. RTW coordinators were not involved in reporting outcomes. Baseline self-report measures were answered by participants before allocation. Researchers were blinded to the identity of participants and each participant’s data had a unique research code.

### Statistical Methods

The independence of study groups was checked with regard to baseline socio-demographic, health and work-related characteristics of participants. Descriptive data are provided for baseline measures and for the cost and content of the intervention. Generalized estimated equations (GEE) regression analysis was performed to analyze the dichotomous outcome variable (≥1 day of competitive work per week) using repeated measurements, an unstructured working correlation structure and treating time as a categorical variable. The first 8-week period following randomization was used as reference category. Precision was measured with 95% confidence intervals (CI). Principles of intention-to-treat analysis were adhered to. Subgroup analysis was performed for defined RTW predictors and factors of specific societal interest to the project: gender, age, educational level, benefit, employment status and diagnosis of sickness certification (Online Resource 1 and 2). Data analysis was performed using STATA version 14.2 (StataCorp. 2015. College Station, Texas, USA).

## Results

### Participant Flow

**Fig. 1:**
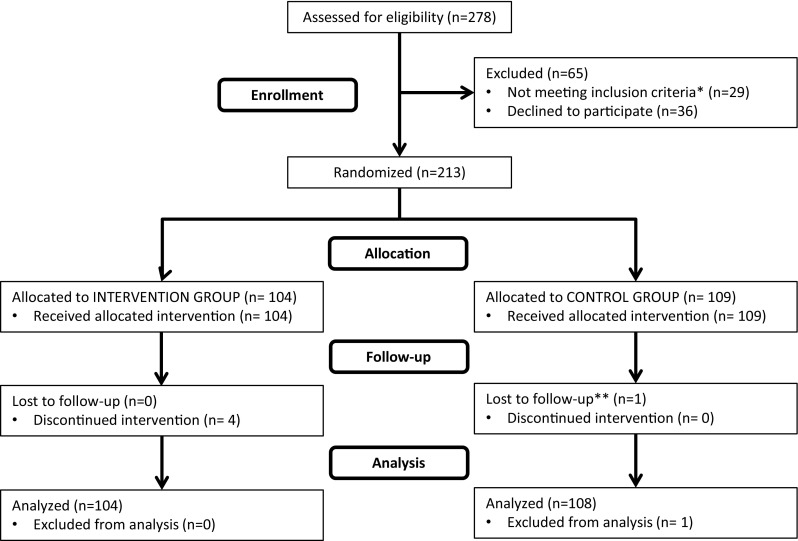
Flow of participants in the study.* Single asterisk* represents not eligible due to: pregnancy (1); incomplete study registration procedure (3); students, i.e. goal of returning to studies rather than to work (6); not registered as receiving temporary medical benefits (10); did not complete rehabilitation program due to acute illness/injury (3); or personal/family reasons (6). Basic baseline characteristics of the nine participants who did not complete the program were checked and found non-discernable from the rest of the sample.* Double asterisk* lost to follow-up only for primary outcome (not secondary)

All 278 participants admitted to the on-site rehabilitation program were assessed for eligibility. Of these 29 patients were not eligible and 36 patients declined to participate. This left 213 participants, of which 104 were randomized to the intervention and the remaining 109 to the control group. Figure 1 shows the flow of participants through the study.

### Lost to Follow-Up

Norwegian Labour and Welfare Service data were available for all participants, but for one participant data on the primary outcome were missing. Overall 2.7% of single longitudinal measurements on primary outcome were missing, evenly spread over time points.

### Baseline Characteristics

A summary of baseline characteristics comparing the intervention and control group is presented in Table 1. Meaningful differences were not found between the two groups when baseline differences were assessed.

**Table 1:** Baseline characteristics of sick-listed participants prior to entering an occupational rehabilitation program

Variables	Intervention	N	Control	N	p value
	n (%) or mean (SD)		n (%) or mean (SD)		
Sociodemography					
Male gender (%)	23 (22%)	104	20 (18%)	109	0.494
Age (mean, SD)	42.9 (0.9)	104	41.7 (0.9)	109	0.349
Higher education (%)	40 (38%)	104	49 (45%)	109	0.337
Health					
Chronic pain^a^ (%)	81 (78%)	104	78 (72%)	108	0.341
Chronic fatigue^b^ (%)	81 (79%)	103	86 (79%)	109	0.958
Mental distress ^c^ (%)	66 (64%)	103	66 (61%)	109	0.596
Comorbidity^d^ (%)	82 (79%)	104	83 (76%)	109	0.637
Sleep disturbance^e^ (%)	54 (52%)	104	52 (48%)	109	0.538
Mental disorder^f^ (%)	36 (38%)	96	38 (37%)	102	0.972
Self-reported disability^g^					
Walking/standing (%)	21 (21%)	101	28 (26%)	107	0.361
Holding/picking up things (%)	18 (18%)	102	14 (13%)	108	0.345
Lifting/carrying (%)	None	101	None	107	
Sitting (%)	15 (15%)	102	20 (19%)	108	0.459
Managing/coping (%)	62 (61%)	102	70 (65%)	107	0.487
Cooperation/communication (%)	37 (36%)	102	48 (45%)	107	0.207
Senses: sight and hearing (%)	16 (16%)	101	15 (14%)	107	0.712
Work and benefits					
Unemployed (%)	47 (43%)	104	39 (38%)	109	0.403
Work assessment allowance^h^ (%)	55 (53%)	104	65 (60%)	109	0.321
Never been/over 3 years since in work	16 (15%)	104	15 (14%)	109	0.737
Combination of benefit and work (%)	35 (34%)	104	36 (33%)	109	0.923
Working 1 day or more per week (%)	30 (29%)	103	32 (30%)	108	0.936
Work participation, hours per week (mean, SD)	4.5 (7.45)	103	4.5 (7.44)	108	0.947

^a^Chronic pain: a score ≥ moderate to very severe pain and duration of ≥6 months

^b^Chronic fatigue: a score of ≥4 on the Chalder Fatigue Scale and duration of ≥6 months

^c^Mental distress: a score of ≥8 on either the anxiety and/or the depressive scales of the HADS

^d^Comorbidity: a combination of ≥2 of the following: chronic fatigue, chronic pain and/or mental distress as defined above in superscript a, b, c

^e^Sleep disturbance: a score of ≥11 on the Insomnia Severity Index

^f^Mental disorder as main diagnosis for sickness certification by the treating doctor, usually the general practitioner. Classified as category for “psychological symptoms/complaints/diagnosis” (P) in the International Classification of Primary Care (ICPC). Remaining participants were classified in other categories (musculoskeletal, general, neurological, etc.)

^g^Self-reported disability: a score ≥3 (moderate disability to more) on the Norwegian Function Assessment Scale (NFAS). Each of the seven functional domains (walking/standing, etc.) are reported separately

^h^Work assessment allowance is a temporary medical benefit granted to individuals who have either been on sickness benefit over 1 year or who due to lack of previous employment do not qualify for sickness benefit. The remaining participants were on sickness benefit

#### Sociodemographic Data

The average age of participants upon entry to the program was 42 years (range 20–59). 170 (80%) were women. 89 (42%) of the participants had higher education (completed college/university education).

#### Symptoms and Diagnoses

Participants reported clinically significant symptom levels as follows: 159 (75%) had chronic pain (of at least moderate intensity); 167 (79%) reported chronic fatigue; and 132 (62%) mental distress. Overlap of these conditions existed for 166 participants (80%). The main medical causes of sick leave specified on the medical certificates were: mental disorder (38%); musculoskeletal disease (30%); general and unspecified disease (20%); neurological (7%). The remaining 5% belonged to a broad diagnostic category including digestive, respiratory, dermatological, reproductive and endocrine diseases.

#### Work and Medical Benefits

All participants were on temporary medical benefits; 93 (44%) received sickness benefits and 120 (56%) work assessment allowance. Only 127 (60%) were in registered employment prior to admission, and 31 (15%) participants self-reported that it was more than 3 years since they had been in work (n = 28) or that they had never been in work (n = 3). Graded work combined with receiving a graded temporary medical benefit was seen for 71 (33%) participants. Average work participation was 4.5 h per week (mode = 0, median = 0, range 0–31 h per week). A total of 62 (29%) participants worked at least 1 day per week on average.

### Primary Outcomes

**Fig. 2:**
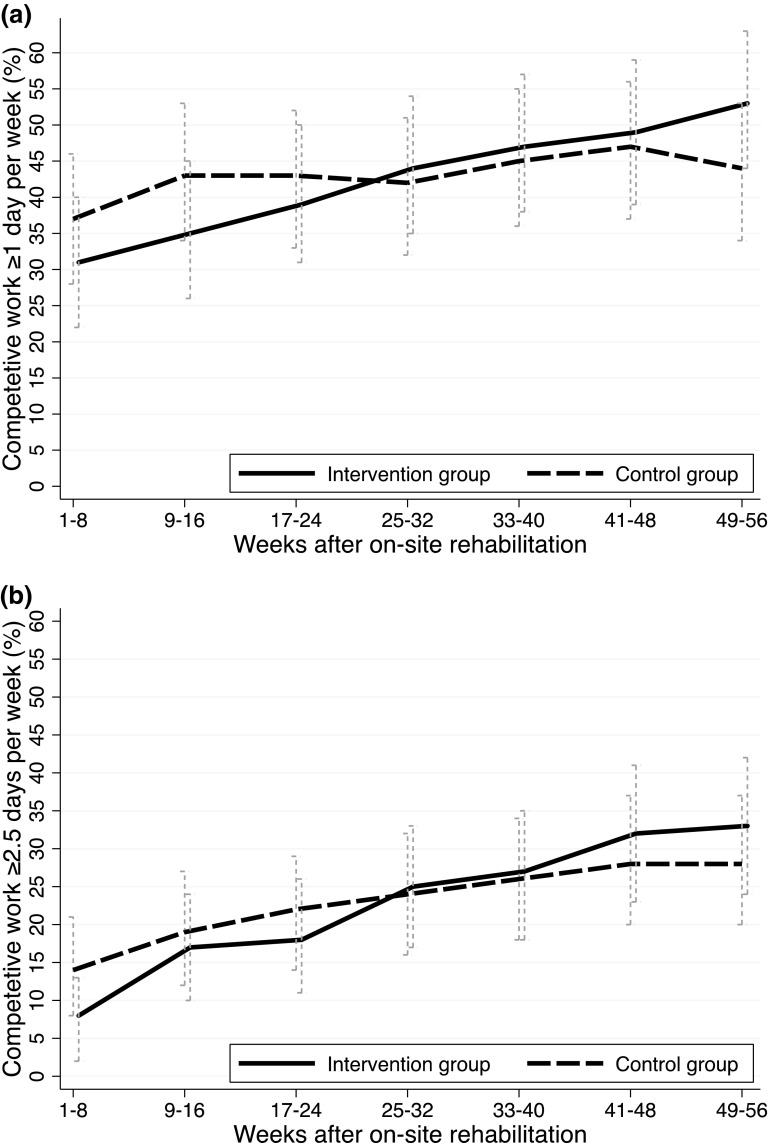
Generalized estimating equations (GEE) analysis of work participation in the intervention and control group during the first year after completing on-site occupational rehabilitation. **a** Main analysis estimated percentages (with 95% confidence intervals) of participants working ≥1 day (7.5 h) per week on average over 8-week periods. **b** Sensitivity analysis estimated percentages (with 95% confidence intervals) of participants working minimum halftime ≥2.5 days (18.75 h) per week on average over 8-week periods

The results from the GEE logistic regression model on the primary outcome are presented in Fig. 2. The intervention group had a linear increase in (re)entry to work over time. For the first 8-week period the control group had higher (re)entry to one day or more of work per week, but their curve flattened out. The intervention group surpassed the control group after 6 months and from that point on the intervention group saw a steadily higher number of participants working 1 day or more per week. The odds of having reentered work were higher for the intervention group compared to the control group (OR 1.87, 95% CI 1.06–3.31, p = 0.031). Sensitivity analysis (GEE) for the cut off “minimum half-time work” and “full-time work” both showed similar patterns to the main analysis. Results from subgroup analysis are included in the Online Resource 1 and 2.

**Fig. 3:**
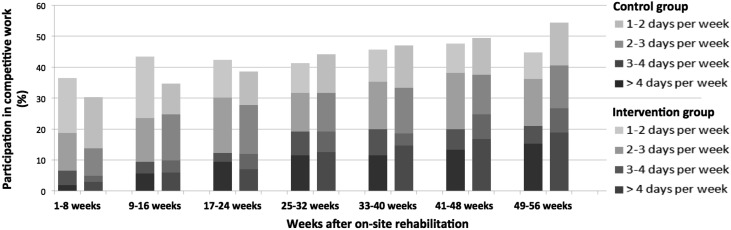
Participation in competitive work for the intervention group (boosted RTW follow-up) versus the control group (standard RTW follow-up). *Bars* show proportion of participants that worked ≥1 day per week on average over 8-week periods during the first year after completing on-site occupational rehabilitation. Shading differentiates according to the average number of days worked per week. (Color figure online)

A larger proportion of the intervention versus the control group had returned to competitive work for all of the explored cut offs after 1 year: minimum substantial work of at least 1 day work per week (54.5 vs. 44.8%), half-time work (32.9 vs. 28.1%) and full-time work (18.8 vs. 15.2%). Based on the difference between the intervention and control group at 1 year after discharge, the *number needed to treat* (NNT) was ten persons for the main outcome of minimum substantial work participation, meaning that an additional one in ten participants receiving boosted follow-up managed to cross the threshold from a non-participatory state to competitive work of 1 day or more per week. During the final registration period (week 49–56) participants over main cut-off (≥1 day work per week) were working 3 days more on average per week for all 8 weeks when compared to those under threshold. Further descriptive data on reentry to work are provided in Fig. 3.

The first 8-week period after discharge was divided into biweekly units to check if differences between groups were present directly following discharge (week 1–2) but at this early point there was minimal difference in the proportion of participants working ≥1 day per week betw een the intervention group (31.1%) versus the control group (31.8%).

### Secondary Outcome

#### Days of Paid Work

The mean total number of days worked during the first year following the rehabilitation program was marginally higher for participants in the intervention group (71 days) compared to the control group (68 days).

#### Benefits

After 1 year a higher number of participants in the intervention group (26%) were working and no longer receiving benefits (medical or non-medical) compared to the control group (19%). The majority of participants were still receiving work assessment allowance (intervention 72% vs. control 74%) but the intervention group was to a larger extent combining graded work and partial benefits by working 1 day or more per week (50% for intervention vs. 47% for control).

#### Cost of Boosted RTW Follow-Up

One-hundred and four participants were included in the cost analysis of which four were classified as discontinued intervention indicating that booster sessions had been prematurely stopped before the recommended 6 months. On average 5.77 contacts (mode 6, range 0–15) per participant were provided throughout the 6-month intervention period (Table 2). One-to-one booster contacts between the RTW coordinator and participant accounted for 87% of all contacts. Collaborative contact with stakeholders was provided for 41.4% of participants, and for these on average 1.8 times per participant (mode 1, range 1–9). The mean cost per participant for a 6 month boosted follow-up was 390.5 EU and the extra time spent by RTW coordinators was on average 7 h per participant.

**Table 2:** Duration and number of contacts in boosted RTW follow-up over the six months intervention period. Intervention cost (Euros) per participant

Type of contact	Time spent per contact (h)	Cost in Euros per contact	Mean number of contacts per participant (SD)	Mean cost in Euros per participant (SD)
Individual contact by telephone	1.17	64 €	5.03 (2.12)	321.4 € (135.5)
Collaborative meeting				
By telephone	1.42	78 €	0.49 (1.03)	38.1 € (80.2)
By videoconference	1.42	78 €	0.12 (0.40)	9.0 € (31.2)
In-person	3.00	164 €	0.13 (0.42)	22.1 € (68.9)
Total			5.77 (2.58)	390.5 € (192.0)

## Discussion

This study specifically investigates boosted RTW follow-up by telephone delivered after discharge from specialist health care, thus focusing on the effect of one single component of a comprehensive and generic occupational rehabilitation program. The main finding after 1 year is that participants receiving 6 months of boosted follow-up had higher odds of moving from a non-participatory state to minimum substantial work of one or more days per week on average over 8 weeks when compared to those receiving standard RTW follow-up only. For every ten participants that received 6 months of boosted follow-up, one additional participant regained the ability to work. The cost of boosted follow-up per participant was 390.5 EUR implying that the cost of one individual (re)entering work was 3905 EUR.

These results should be discussed with awareness concerning two factors defining the population and the intervention. First, the occupational rehabilitation program had an open door policy of admitting participants with both mental and somatic disorders, regardless of employment status and without requirement of previous work experience. In contrast, many RTW programs are diagnosis specific, exclude the unemployed or focus exclusively on sick leave of shorter duration. Baseline characteristics revealed a predominantly female population characterized by a high symptom burden, high comorbidity and a weakened connection to the work force seen through a large proportion being unemployed or on medical benefits over several years. Mental disorders were the most common cause of sickness certification. The majority had not completed higher education. According to the standard literature, these characteristics may indicate barriers or a poorer prognosis for (re)entry to the work force and a higher chance of moving on to permanent disability benefits [41–44]. Moreover, the characteristics of the study population mirror the population in Norway on long term sick leave, being the most challenging group for RTW. Studies on marginalized groups are encouraged [45], and recent studies have shown that RTW interventions can be particularly effective for these groups [15].

The second factor is the low dose and hence the limited cost of boosted follow-up. Previous literature in the RTW field has been unclear on how long or how intensive follow-up after occupational rehabilitation programs should be. Boosted follow-up was brief in terms of duration and frequency of contacts. On average 5.8 contacts per participant were delivered over 6 months representing <7 h of extra work for the RTW coordinator and with an extra cost of 390.5 EUR. A standard cost-effectiveness analysis comparing incremental costs and effects within a societal context was not undertaken since limited cost information was available (intervention costs only). We found only a marginally higher total number of days worked during the first year following the rehabilitation program for those receiving boosted follow up, but one in ten participants benefitted from boosted follow up by (re)entering work (above the 1 day or more per week threshold). During the final 8-week registration period the average gain in number of working days seen by those that had (re)entered work was 3 days per week, giving a total of 24 additional days worked in the last period when compared to participants working below threshold. It is important to bear in mind that the participants prior to the boosted follow-up had received a rather costly rehabilitation program, but within this rehabilitation context the results indicate that the use of boosted follow-up should be considered.

The time trajectories for (re)entering competitive work clearly differ for the two groups (see Fig. 2) illustrating the importance of analyzing development of work participation over time [46, 47]. The control group initially returned more rapidly to at least 1 day work per week, but lost momentum and was after 6 months surpassed by the intervention group which showed substantial and steady return to work with still increasing work participation at 1 year. It seems that boosted follow-up supported a more gradual work resumption. Previous studies have discussed that interventions supporting step-wise and partial RTW are more effective at promoting return to work [48–52]. The time lag before effect may reflect that vulnerable groups trying to enter the workforce need time to draw benefit from RTW interventions. A pattern of initial delay followed by increased effect on RTW was also seen in a study on rehabilitation for sick-listed individuals that were unemployed/temporary agency workers [45].

Although both groups showed similar work productivity when looking at the year as a whole, the intervention group had achieved a higher degree of inclusion in the work force after 1 year. A larger proportion of the intervention group were engaged in competitive work at least 1 day per week and were working more days per week, compared to the controls (Fig. 3). Sensitivity analysis confirmed that the intervention group was also to a larger extent working either half- or full-time. This can be viewed as the intervention group having achieved a stronger and more stable connection to the work force and a more advantageous position for further stabilizing and increasing work participation. This positive effect is assumed due to the synergism of combining a comprehensive occupational rehabilitation program with boosted follow-up, thus providing continuation of structure and support in the vulnerable phase of transition to daily life. This view finds support in studies emphasizing boosted follow-up as a means of providing continuity in care and maintaining and reinforcing newly acquired cognitive skills over time [53].

### Limitations of the Study

Firstly, the absence of blinding of participants and coordinators to the nature of the allocation is a limitation. There mays have been an anticipatory effect for participants receiving the intervention and a negative effect on those not allocated to boosted follow-up. Blinding was not feasible, but using registry data ensured that outcome measurements were independent of allocation. Secondly, combining different sources of registry data for the outcome measure (hours worked per week) creates a potential for measurement bias. Since this is a randomized controlled study any inconsistency in the method should equally affect both groups. Thirdly, it can be discussed if participation in work at least 1 day (7.5 h) per week represents a significant improvement in RTW. Productivity is still limited at this level, but employment is feasible and the outcome may be looked at as preparatory to more substantial employment. The cut-off was considered realistic for participants marginalized from the work force and sensitivity analysis for cut-offs at higher levels of employment did not show diverging results. Finally, the cost of introductory training and biweekly group counseling of coordinators was not included in the cost analysis. However, these costs were mainly related to the on-site program, not the intervention. The intervention cost was realistic in the sense that wage cost per hour of clinical work included overhead costs as well as an assumed proportion of time spent on administrative tasks.

### Strengths of the Study

Firstly, the study has high generalizability since it targeted a large group of the sick-listed population (broad diagnostic range, including mental and physical disorders, all grades of sick leave, employed and non-employed). The study includes groups for whom RTW studies have been lacking and provides new knowledge on a more generic approach to occupational rehabilitation. Secondly, high adherence to the intervention protocol was observed for RTW coordinators and participants alike. Thirdly, there was minimal loss to follow-up and very little missing data for both baseline and outcome measures. Finally, this study gives valuable insight into the time trajectory for return to work, which is often lacking in similar studies.

### Implications of the Study

Complex and expensive rehabilitation programs risk being shut down since effects are rarely more than moderate and must be weighed against considerable costs and resources spent delivering programs [54]. In addition to being costly, high intensity case management may show poorer results on RTW than less intensive approaches [55]. This study supports that a simple follow-up method may improve RTW without unnecessarily draining resources. This strengthens the growing evidence-base that clinicians and social insurance organizations need when deciding whether to enrich rehabilitation programs by adding simple follow-up regimes to enhance sustainability [11, 56–58].

Boosted follow-up as used in this study should be adaptable to varying clinical settings since it was delivered by RTW coordinators of various professional backgrounds, did not require additional skills or training beyond that required for delivering the on-site program, was delivered to participants in geographically distant settings and did not demand access to expensive or technically complicated equipment. The overall versatility and low cost of the boosted follow-up regime lowers the barriers for implementing it in clinical practice.

Young adults with health-issues that face problems (re)entering the work force are internationally a matter of high individual, societal and political concern [59]. Some welfare states such as Norway, see rising numbers of young adults on long term health-related benefits [60]. In this study, participants aged 35 years and younger seemed to particularly benefit from boosted follow-up. However, results had low precision and subgroup analysis on small groups should be interpreted with caution. We hypothesize that participants struggling the most to establish themselves in the work force may be those that benefit the greatest from boosted follow-up. Likewise, for groups that face lesser barriers, adding follow-up may not be necessary.

Based on the results from this study boosted follow-up is recommended translated within the field of occupational rehabilitation. The financial risk seems low. Further research should investigate the optimal length of boosted follow-up, how it performs in conjunction with other types of occupational rehabilitation programs and explore possible mechanisms (mediators) of the effect of boosted follow-up, such as for example use of acquired cognitive skills in the daily environment. Studies with larger populations would allow predictive subgroup analysis to see which participants benefit from boosted follow-up while testing repeatability of results. We recommend that studies target groups facing the largest barriers to (re)entering the work force. Studies of longer duration are necessary to check if the observed effect is stable over time. Data up to 5 years post-discharge are being collected in this study.

## Conclusions

Systematic telephone follow-up after discharge from occupational rehabilitation increased work participation for a population at high risk of permanent work disability. One year after randomization, participants having received 6 months of boosted RTW follow-up had substantially higher odds of having moved from non-participation to (re)entering paid work ≥1 day per week when compared to the control group. The added cost of boosted RTW follow-up from the perspective of the occupational rehabilitation institution was 390.5 Euros. We conclude that a small dose of continued support in the transition phase from on-site occupational rehabilitation to (re)entering a working environment may increase work participation at limited cost.

## Electronic supplementary material

Below is the link to the electronic supplementary material.

Online Resource 1 Explanatory text accompanying subgroup analyses. (DOCX 109 KB)

Online Resource 2 Collection of figures 4.-9. Subgroup analysis. Generalized estimating equations (GEE) analysis of work participation in the intervention and control group during the first year after completing on-site occupational rehabilitation. (PDF 172 KB)

## Footnotes

1 Norwegian Central Bank, January 2014.
