# Supplementary material for: Randomized Controlled Trial of Adding Telephone Follow-Up to an Occupational Rehabilitation Program to Increase Work Participation
Source: J Occup Rehabil. 2017 Jun 9;28(2):265–78. doi: 10.1007/s10926-017-9711-4 (PMC5978834; doi:10.1007/s10926-017-9711-4)
Supplement: Supplementary file 1 — Online Resource 1 Explanatory text accompanying subgroup analyses. (DOCX 109 KB) [file 10926_2017_9711_MOESM1_ESM.docx]

# Online Resource 1: Text accompanying subgroup analyses

# Randomized controlled trial of adding telephone follow-up to an occupational rehabilitation program to increase work participation.

Karen Walseth Hara MD^1, 2, 3, 4^, Johan Håkon Bjørngaard PhD^1, 5^, Søren Brage MD, PhD^6^, Petter Christian Borchgrevink MD, PhD^2, 3,7^, Vidar Halsteinli PhD^1, 8^, Tore Charles Stiles PhD^9^, Roar Johnsen PhD^1^, Astrid Woodhouse PhD^1, 2^

^1^Department of Public Health and Nursing, Faculty of Medicine and Health Sciences, Norwegian University of Science and Technology (NTNU), Trondheim, Norway

^2^Norwegian Advisory Unit on Complex Symptom Disorders, St. Olavs Hospital, Trondheim University Hospital, Trondheim, Norway

^3^Department of Circulation and Medical Imaging, Faculty of Medicine and Heath Sciences, Norwegian University of Science and Technology (NTNU), Trondheim, Norway

^4^ The Norwegian Labour and Welfare Service of Sør-Trøndelag, Trondheim, Norway

^5^ Forensic Department and Research Centre Brøset, St. Olavs Hospital, Trondheim University Hospital, Trondheim, Norway

^6^ The Norwegian Directorate for Labour and Welfare, Oslo, Norway

^7^Hysnes Rehabilitation Center, St. Olavs Hospital, Trondheim University Hospital, Trondheim Norway

^8^Centre for Health Care Improvement, St. Olavs Hospital, Trondheim University Hospital, Trondheim, Norway

^9^Department of Psychology, Faculty of Social and Educational Sciences, Norwegian University of Science and Technology (NTNU), Trondheim, Norway

**Corresponding Author:**

Karen Walseth Hara

Norwegian University of Science and Technology (NTNU)

Faculty of Medicine and Health Sciences, Department of Public Health and Nursing

Postbox 8905, 7491 Trondheim, Norway

Phone: + 47 93016098

Fax: + 47 73597577

## Method

Analyses were performed to examine the heterogeneity of trajectories for RTW for subsets of participants. Predictors of RTW and factors of special societal interest had been identified pre-trial when designing the larger research project. Subgroup analysis is restricted to these, and further narrowed down to those factors that we had pre-admission data on. Similar to the main analysis we performed generalized estimating equations (GEE) analysis of the time trajectory of work participation during the first year after completing on-site occupational rehabilitation for the outcome ≥ 1 day of competitive work per week over 8 week periods. Subgroups comprising less than 20% of the study population were not analyzed due to small size of the subgroup. The following baseline variables were analyzed:

- *Gender* (analyzed only for females as males comprised < 20% of the study population)
- *Age ≥ 36 years old*. This is the legislative cut-off used in the Norwegian social security system when applying for special disability benefits for young people with regard to assessing work related activity.
- *Higher education*, i.e. completed college/university education *(yes/no)*
- *Benefit (sickness benefit/work assessment allowance)*
- *Employment status* (employed/unemployed)
- *Diagnosis on sickness certification* (somatic/mental disorder)

## Comments:

The results from the GEE logistic regression model on the primary outcome are presented in figures 4. – 9. (Online Resource 2) for the analyzed subgroups. Substantial differences at subgroup level are not seen between intervention and control groups for the odds of having reentered work over time. Notably, despite low precision, for the fifty-eight (27%) participants aged 35 years and younger, the odds of having reentered work were 184% higher for the intervention group compared to the control group (OR 2.84, 95% CI 0.868 – 9.292, p= 0.084). This is briefly discussed in the main text under implications.
