# Supplementary material for: Randomized Controlled Trial of Adding Telephone Follow-Up to an Occupational Rehabilitation Program to Increase Work Participation
Source: J Occup Rehabil. 2017 Jun 9;28(2):265–78. doi: 10.1007/s10926-017-9711-4 (PMC5978834; doi:10.1007/s10926-017-9711-4)

Online Resource 2: Text accompanying subgroup analyses

Randomized controlled trial of adding telephone follow-up to an occupational rehabilitation program to increase work participation.

Karen Walseth Hara MD[1, 2, 3, 4,] Johan Håkon Bjørngaard PhD[1, 5,] Søren Brage MD, PhD[6], Petter Christian Borchgrevink MD, PhD[2, 3, 7], Vidar Halsteinli PhD[1, 8], Tore Charles Stiles PhD[9], Roar Johnsen PhD[1], Astrid Woodhouse PhD[1, 2, 3]

[1] Department of Public Health and Nursing, Faculty of Medicine and Health Sciences, Norwegian University of Science and Technology (NTNU), Trondheim, Norway
[2] Norwegian Advisory Unit on Complex Symptom Disorders, St. Olavs Hospital, Trondheim University Hospital, Trondheim, Norway
[3] Department of Circulation and Medical Imaging, Faculty of Medicine and Health Sciences, Norwegian University of Science and Technology (NTNU), Trondheim, Norway
[4] The Norwegian Labour and Welfare Service of Sør-Trøndelag, Trondheim, Norway
[5] Forensic Department and Research Centre Brøset, St. Olavs Hospital, Trondheim University Hospital, Trondheim, Norway
[6] The Norwegian Directorate for Labour and Welfare, Oslo, Norway
[7] Hysnes Rehabilitation Center, St. Olavs Hospital, Trondheim University Hospital, Trondheim Norway
[8] Centre for Health Care Improvement, St. Olavs Hospital, Trondheim University Hospital, Trondheim, Norway
[9] Department of Psychology, Faculty of Social and Educational Sciences, Norwegian University of Science and Technology (NTNU), Trondheim, Norway

Corresponding Author:
Karen Walseth Hara
Norwegian University of Science and Technology (NTNU)
Faculty of Medicine and Health Sciences, Department of Public Health and Nursing
Postbox 8905, 7491 Trondheim, Norway
Phone: + 47 93016098
Fax: + 47 73597577

Demography

Figure 4.

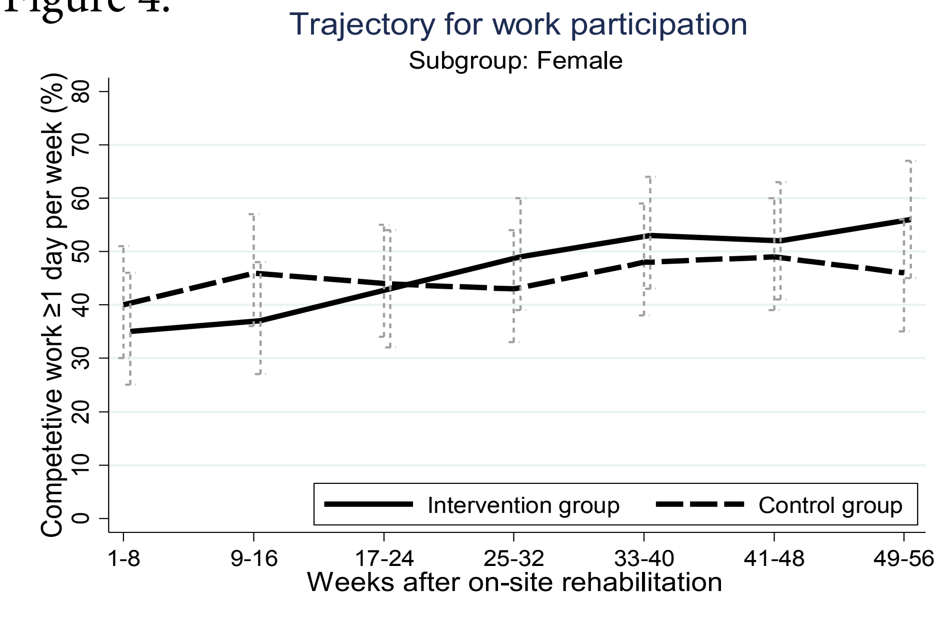

Figure 5a.

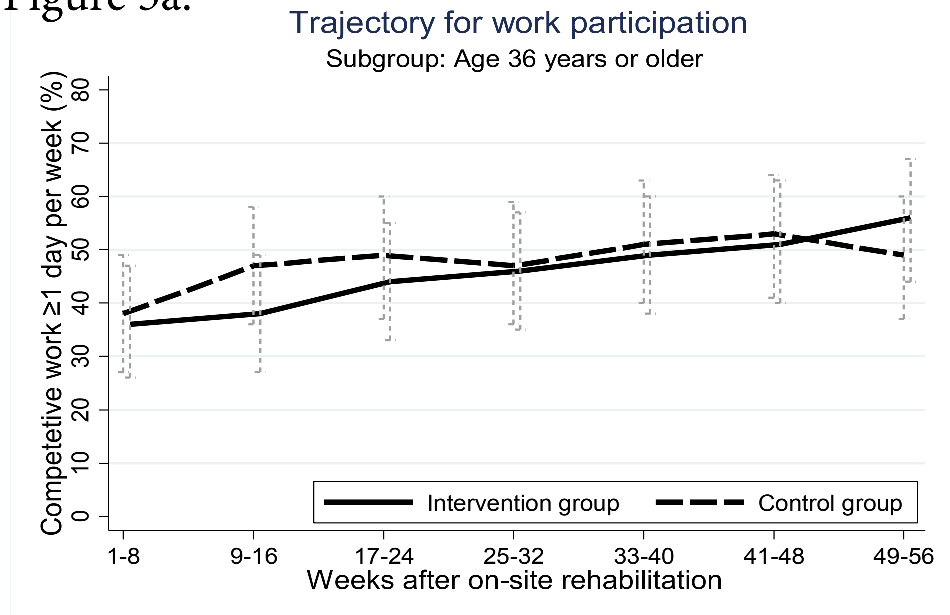

Figure 5b.

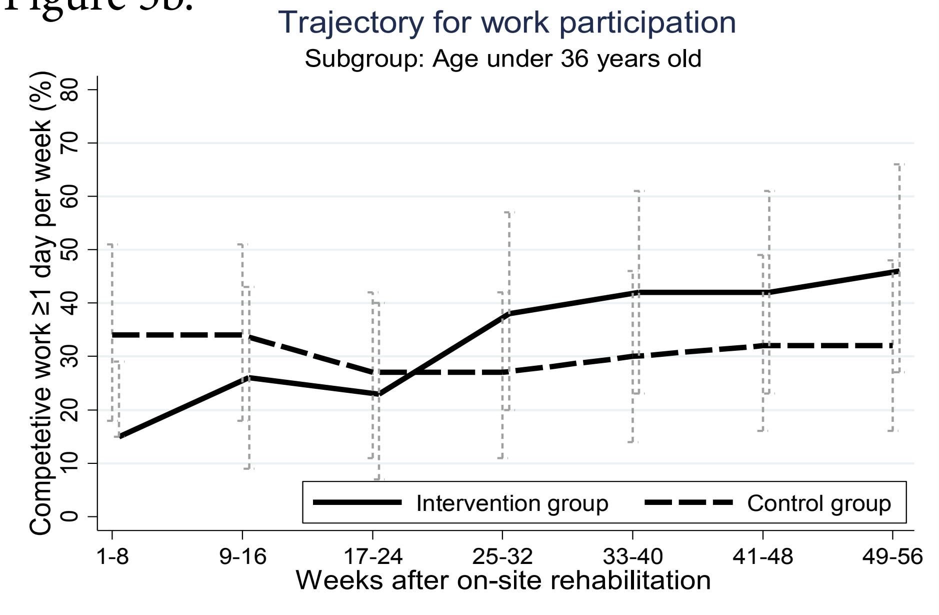

Demography

Figure 6a.

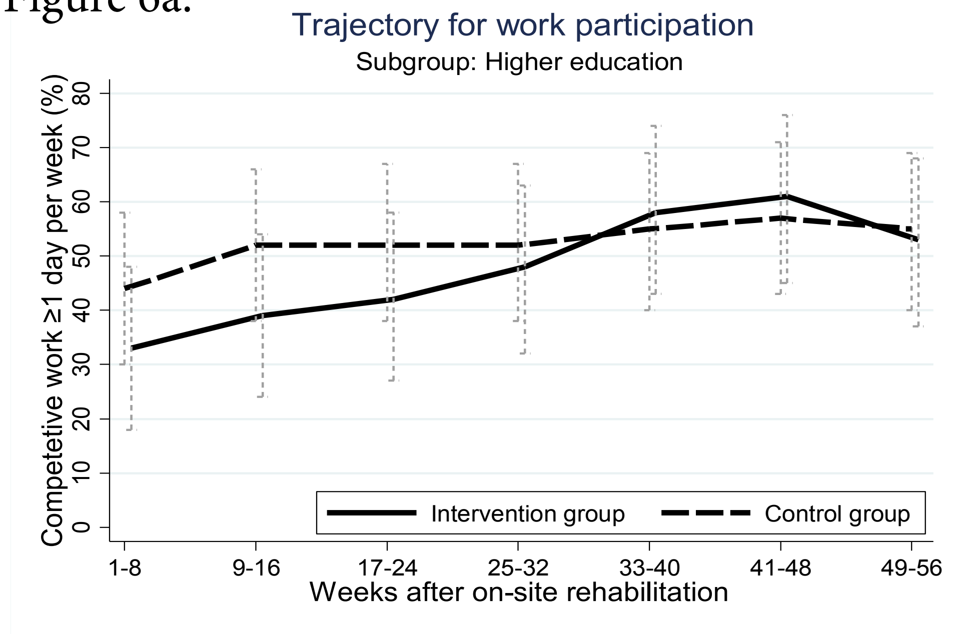

Figure 6b.

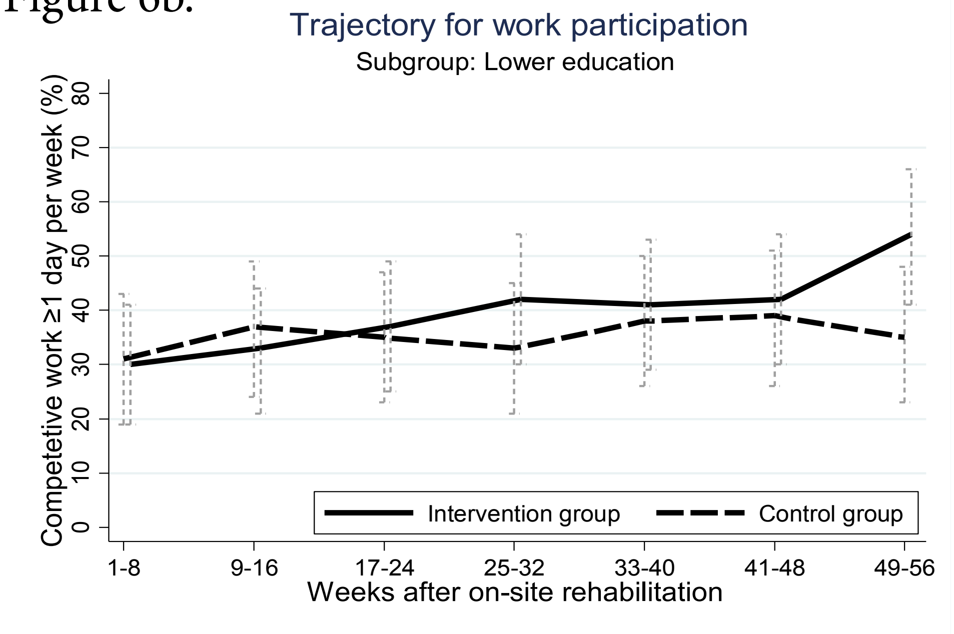

Work and benefits

Figure 7a.

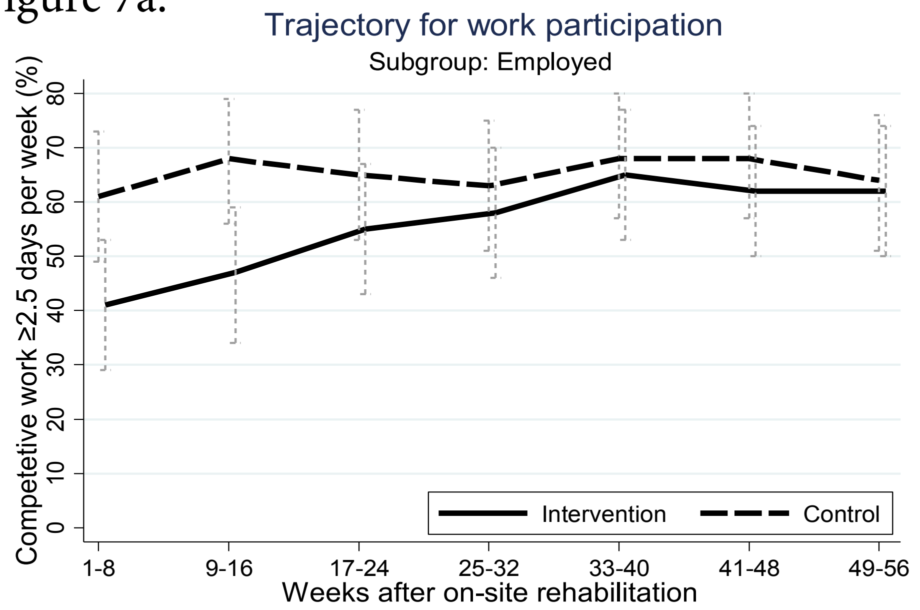

Figure 7b.

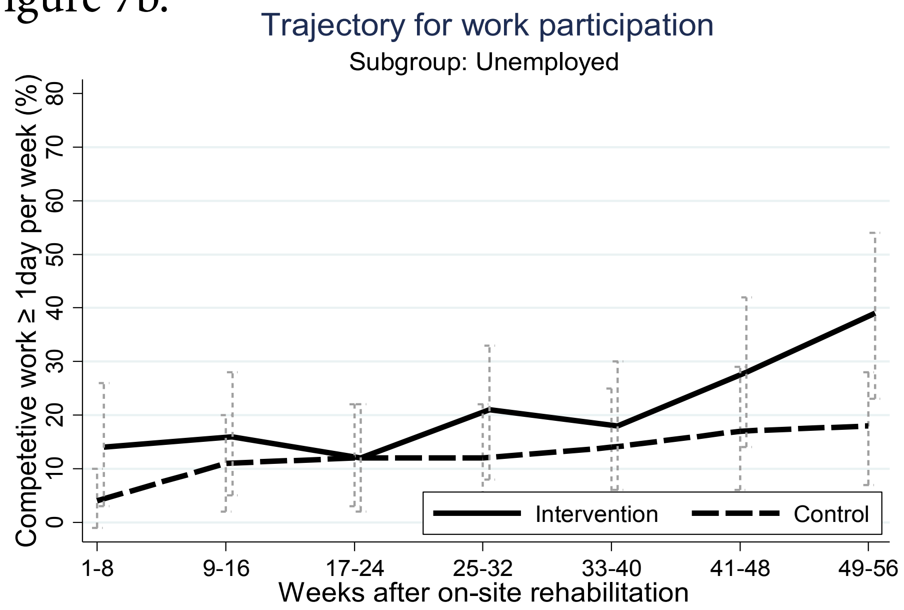

Figure 8a.

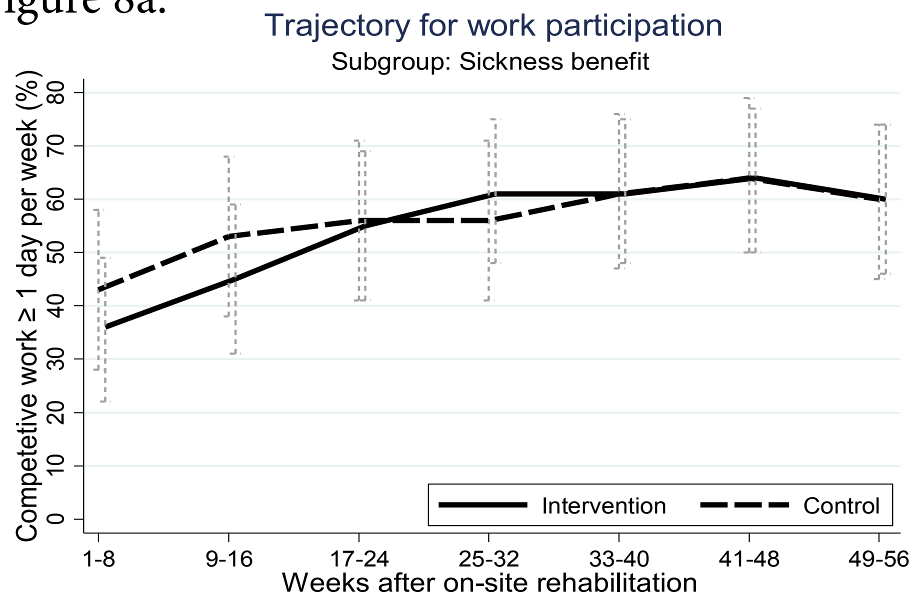

Figure 8b.

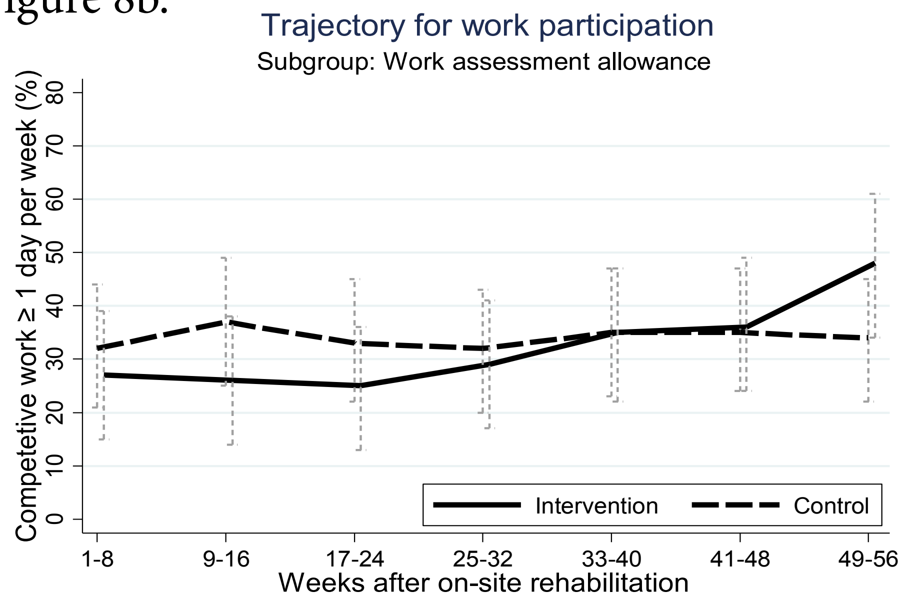

Health related factors

Figure 9a.

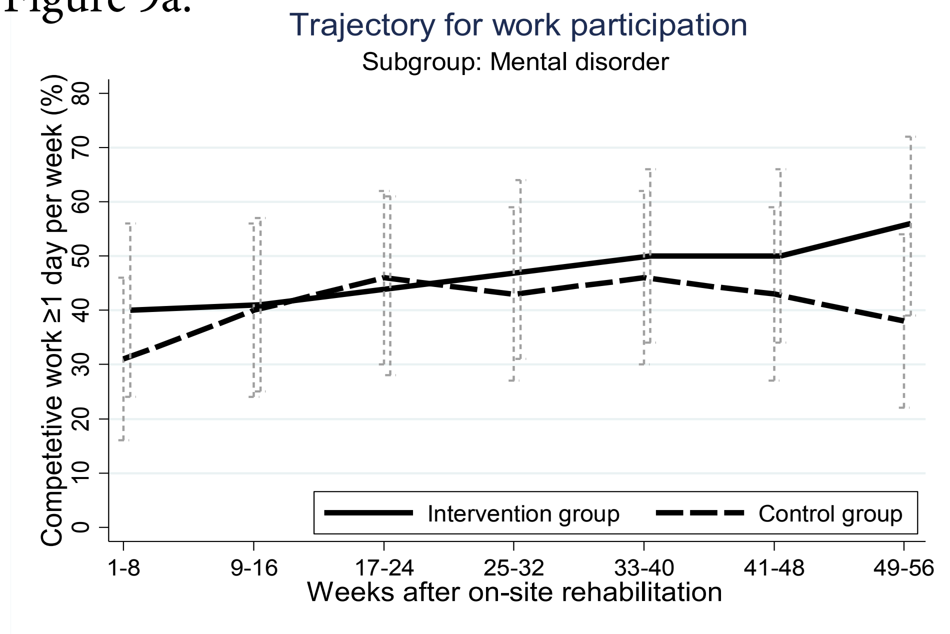

Figure 9b.

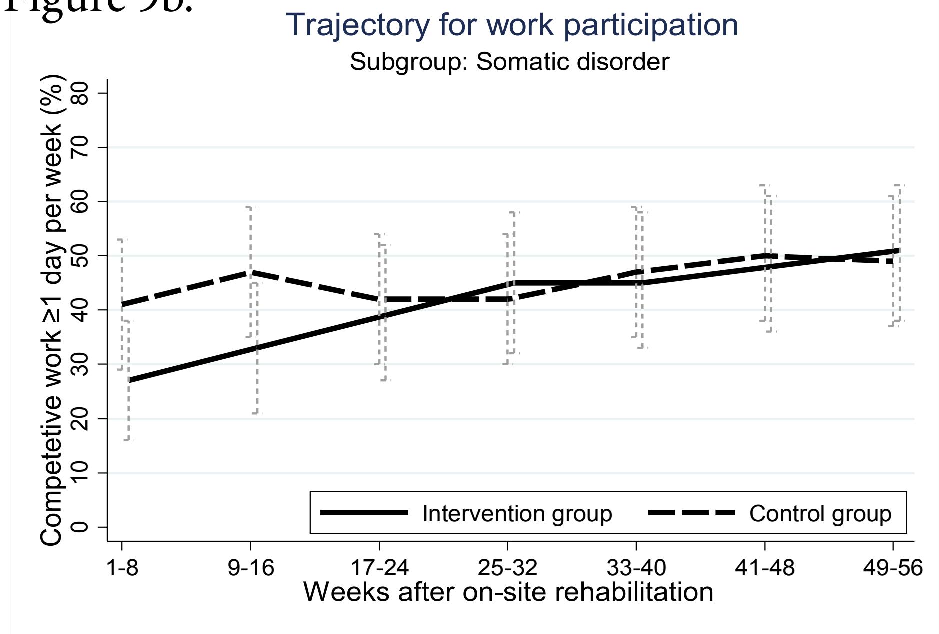

Supplement: Supplementary file 2 — Online Resource 2 Collection of figures 4.-9. Subgroup analysis. Generalized estimating equations (GEE) analysis of work participation in the intervention and control group during the first year after completing on-site occupational rehabilitation. (PDF 172 KB) [file 10926_2017_9711_MOESM2_ESM.pdf]
